# How perceptual differences between leaders and followers affect the resilience-workability relationship

**DOI:** 10.3389/fpsyg.2022.1066909

**Published:** 2023-01-09

**Authors:** Marjolein C. J. Caniëls

**Affiliations:** Faculty of Management, Open Universiteit, Heerlen, Netherlands

**Keywords:** resilience, workability, influence tactics, rational persuasion, pressure, two-waved, dyads

## Abstract

Drawing on Conservation of Resources theory and its notion of resource passageways, the aim of this study is to investigate the relationship between resilience and workability, and particularly the extent in which this relationship is buffered or strengthened by differences in perception between leaders and employees about the degree in which a certain influence tactic (pressure or rational persuasion) is used. To this end, this study uses a two-wave time-lagged survey design with a multi-sourced sample of 146 leader-follower dyads. Findings indicate that leader-follower perceptual differences about the use of pressure as an influence tactic buffers the positive resilience-workability relationship of followers. No evidence was found of a similar effect with respect to rational persuasion as an influence tactic.

## 1. Introduction

Over the past years studies have emphasized the importance of resilience at the workplace (e.g., Kuntz et al., 2016; Caniëls and Hatak, 2019; Näswall et al., 2019). Resilience refers to the capability to bounce back from adversity and adapt to changing circumstances and setbacks (Näswall et al., 2019). Resilient employees are expected to display high levels of workability (Semeijn et al., 2019). Workability is defined as the extent to which employees positively assess their ability to work at their current employer (Ilmarinen et al., 2005). Studies have shown that resilience is positively related to health (Smith et al., 2008; Papazoglou and Andersen, 2014) and health is known to positively affect workability (Van den Berg et al., 2009). The idea that resilience is beneficial for workability is in line with Conservation of Resources theory (COR; Hobfoll, 1989). COR theory states that employees with resources - such as the personal resource of resilience - have more possibilities to invest these resources to gather new resources, than employees who lack the initial resources. Hence, drawing on COR theory, it is likely that resilient employees (as opposed to less resilient employees) have more opportunities to conserve resources and invest them, thereby increasing their workability.

Despite its importance, the relationship between resilience and workability has only scarcely been studied. A cross-sectional study among 44 breast cancer survivors (Gómez-Molinero et al., 2019) showed a significant positive association between a global resilience measure and workability, while in a study among police officers (Semeijn et al., 2019) a resilience-workability relationship could not be established due to technicalities in the model assessment. Consequently, little is known about the resilience-workability relationship among office workers. Even less is known about how organizational supportive factors influence this resilience-workability relationship. Examining these supportive factors, i.e., the role of boundary conditions, for this relationship is especially valuable, because it can help organizations direct their efforts toward improving employees’ workability, given their resilience.

With respect to factors that could be supportive for the resilience-workability relationship, COR theory proposes the presence of passageways, i.e., organizational conditions that may accelerate or suppress a change in resources (Hobfoll, 2001, 2011; Halbesleben et al., 2014). Such passageways can manifest themselves in the form of influence strategies, also labeled influence tactics, that are used by the leader to influence the attitude and behavior of employees (Yukl et al., 2005), such as pressure or rational persuasion. In terms of COR theory, influence tactics may encourage or thwart the conservation of resources by employees, thereby leading to either positive, or poor outcomes.

In prior studies, leadership influence tactics are usually assessed solely from an employee perspective (Yukl et al., 2005). When employees experience undesired pressure from leaders, they are likely to try to change this behavior either by speaking up (e.g., during yearly development and assessment talks) or by exerting less overt influence tactics themselves. A problem occurs when the leader does not recognize that he/she puts pressure on an employee. In this situation there is no opportunity to arrive at a resolution of the issue. By solely focusing on employee ratings of leadership influence strategies, studies have missed the opportunity to include an additional perspective on the used influence strategies, namely the perspective of the leader. It may be so that leaders and employees vastly differ in their perspective on the used influence strategy. Such a difference in perspective may have an important boundary effect on the resilience-workability relationship, because a leader, who does not recognize his/her influence strategy in the same way as is perceived by the employee, will not be open to changing his/her behavior. Therefore, it may not be so much the issue whether an employee experiences pressure from a leader, but more so if this leader does not realize that he/she puts pressure on the employee. Differences in perception between leaders and followers are therefore expected to influence the resilience-workability relationship of followers.

The aim of this study is to investigate the relationship between resilience and workability, as well as the extent in which this relationship is buffered or strengthened by differences in perception between leaders and followers about the degree in which a certain influence strategy (pressure or rational persuasion) is used. To this end, this study uses a two-wave time-lagged study design with a multi-sourced sample of 146 leader-follower dyads. The present study aims to answer two main research questions: (1) to what extent is resilience related to workability, and (2) to what extent is the resilience-workability relationship affected by leader-follower perceptual differences in the use of influence tactics (i.e., pressure and rational persuasion).

This study presents one of the first empirical attempts to explore the boundary conditions that determine whether and to what extent resilient employee behavior is related to employee workability. In addition, the current study is one of the first that explores differences in perception between leaders and their followers about the tactics used by leaders. Therefore, our study harbors several theoretical contributions. First, it responds to calls for investigating the role of passageways (Halbesleben et al., 2014), which until now has received scant attention in the literature. By exploring the organizational conditions that may accelerate or hamper the conservation of resources by employees, a gap is filled in empirical studies that until now have mainly focused on studying how personal resources are invested to hedge against resource losses as well as overcoming them (Halbesleben et al., 2014). Second, the current study explores assessment differences between leaders and their followers regarding the degree in which certain influence tactics are used. Consequently, this study advances current knowledge about whether and how differences in perspective between leaders and followers may act as a passageway and hence function as an important boundary effect on the resilience-workability relationship. In this way, this study offers a nuanced understanding of the relationship between resilience and workability.

## 2. Literature review and hypothesis development

Conservation of Resources theory (COR; Hobfoll, 1989) is particularly relevant when studying the relationship between personal resources and workability. Employees use personal resources for self-regulation, for conducting social relations, and for carrying out work tasks (Hobfoll, 2011), in other words, they use personal resources to improve their workability. COR theory poses that individuals are motivated to shield their resources, to use them sparingly as not to deplete them, and to be constantly on the lookout for obtaining new and more resources (Halbesleben et al., 2014). It suggests that when individuals encounter challenges, for instance at work, they try to conserve their personal resources to protect themselves and to meet the demands of the situation at hand. In a work context, personal resources can be conceived of all personal assets that are of value to an employee, e.g., ability, self-esteem and self-efficacy (Hobfoll, 2011, p. 117). Resilience, i.e., having the capacity to be resilient, is also such a personal resource. It can be seen as a means of protecting other resources, and as a way of accumulating additional resources for the future.

### 2.1. Resilience and workability

Luthans et al. (2007) identified resilience as a ‘psychological capital’ resource (Upadyaya et al., 2016). It may help employees to cope with job requirements and therefore support them to conserve resources. Resilient employees are able to recover from setbacks and even thrive in the face of adversity (Smith et al., 2008). Resilience can be nurtured and it can be gradually improved over time (Luthans, 2002; Xanthopoulou et al., 2009; Caniëls and Baaten, 2019), for instance by specifically designed interventions (Bardoel et al., 2014), such as developmental training targeted at the construction of social identities (Roberts and Wood, 2006; Lodi-Smith and Roberts, 2007).

Having the capacity to be resilient provides several advantages in a workplace setting (Kuntz et al., 2016; Caniëls and Hatak, 2019; Näswall et al., 2019). Being able to cope with failure and setbacks improves chances of employees for excelling in their work. Furthermore, resilient employees signal to their organization that they are committed to organizational goals even under conditions of stress and change (Coutu, 2002; Caniëls and Baaten, 2019). Personal resources, such as resilience, have been shown to be crucial for functioning at work (Luthans et al., 2007; Van Dam and Shannon, 2013). Moreover, prior research has positively linked resilience to job performance (Peterson et al., 2011), organizational commitment (Youssef and Luthans, 2007; Meneghel et al., 2016; Wang et al., 2017), thriving at work (Paterson et al., 2014), and overall well-being (Roche et al., 2014; Siu et al., 2015).

Resilience is also likely to be positively associated with the ability to work, i.e., workability (Ilmarinen et al., 2005; Semeijn et al., 2019), as prior studies have shown that resilience is positively associated with closely linked indicators, such as employability and vitality (Avey et al., 2009, 2011; Semeijn et al., 2019). Resilient employees feel confident that they can overcome difficult situations at work and therefore are likely to positively assess their ability to work.

*Hypothesis 1*: Resilience is positively associated with workability.

### 2.2. Influence strategies and leader-employee assessment differences

Leaders influence their followers to carry out their requests. It is essential for leaders to employ an array of influence tactics to ensure the performance of their followers (Yukl et al., 2008). Different tactics are suitable for different objectives. For example, influence tactics that relate to impression management are used by leaders to build and improve their relationship with their followers (e.g., Kumar and Beyerlein, 1991). In contrast, proactive influence tactics are used by leaders in an attempt to influence followers to undertake immediate action and tend to a certain request (Yukl et al., 2008). Exerting pressure and using rational persuasion are two tactics that fall into this latter category. Leaders who employ pressure as an influence tactic, use persistent reminders, threats and/or insistent demands to influence their followers to carry out an immediate request. When rational persuasion is utilized, leaders make use of logical arguments and factual evidence to convince followers of the importance of carrying out the immediate request (Yukl et al., 2008).

When a tactic is used for a legitimate request and when the tactic is in line with a follower’s values and needs, it is likely that the tactic will be successful and the follower will carry out the request (Yukl et al., 2008). Therefore, tactics that appeal to rational arguments and evidence are more likely to receive compliance than tactics that make use of manipulation, coercion and pressure. To determine whether leadership influence tactics are successful, they are usually evaluated from the perspective of either the leader (Kacmar et al., 2013; Curtis, 2018) or the employee (e.g., Yukl et al., 2005; Clarke and Ward, 2006). However, when leaders are asked to assess their own influence tactics, it is likely that socially desirable ratings are provided. Following this reasoning, it seems more apt to evaluate how followers assess the influence tactics that they experience. Furthermore, an assessment of the leader’s preferred tactic by followers may be of practical use within a work context. In a workplace setting, followers have certain role expectations regarding their leader, i.e., beliefs about acts that leaders should or should not display (Katz and Kahn, 1966; Wong, 2019). Followers have been found to engage in upward influence behaviors when leaders’ behavior deviates from the followers’ beliefs about how they should be treated by their leader (Wong, 2019). Hence, it is to be expected that when an employee experiences negative tactics from a leader, such as pressure, employees will engage in influencing behavior to correct this deviation from desired leader behavior. In addition, the issue will be discussed during yearly development and assessment talks. Leaders may reflect upon their behavior and how it is perceived by their followers and consequently adapt it.

A problem occurs when leaders do not acknowledge or recognize that they put pressure on their employees. In this situation, a gap emerges between what leaders acknowledge that they are doing and what followers experience. Drawing on COR theory (Hobfoll, 2011) and especially on the concept of ‘resource caravan passageways’ (Hobfoll, 2011, 2012), we argue that leader-follower differences in the assessment of leader pressure may act as a resource passageway. The concept of resource passageways refers to organizational “environmental conditions that support, foster, enrich, and protect the resources of individuals, sections or segments of workers, and organizations in total, or that detract, undermine, obstruct, or impoverish people’s or group’s resource reservoirs” (Hobfoll, 2011, p. 119). We argue that leader-follower assessment differences of leader pressure may function as a resource passageway and exert an important boundary effect on the resilience-workability relationship. The assessment differences may suppress and deplete followers’ resources (i.e., resilience) and thwart the possibility to obtain new resources (i.e., improved workability).

In situations where leaders and employees do not differ in their perception of the used influence strategy we expect a positive relationship between resilience and workability. However, in situations where differences between leaders’ and employees’ perspectives are large, i.e., followers experience more pressure than leaders perceive that they exert, the positive relationship between resilience and workability will be buffered. This is because followers may feel that their fundamental norms of respect and understanding are violated (Kane and Montgomery, 1998), which depletes resources and thwarts the acquirement of resources.

Additionally, one could conceive of a situation where employees asses their leader as less pressuring than the leader assesses him/herself. In such cases, drawing on the principle of resources passageways, it is expected that the possibility to use followers’ resources (resilience) for obtaining more resources (workability) is complemented, as followers do not experience thwarting by the leader’s use of pressure. Hence, the positive resilience-workability relationship is strengthened. Taken altogether, the following is hypothesized:

*Hypothesis 2*: Leader-employee differences in the assessment of leader pressure moderates the positive relationship between resilience and workability in such a way that this relationship is buffered when followers experience more pressure than leaders perceive that they exert, and strengthened when follower ratings of leader pressure are lower than leader ratings.

Similarly, a gap between leaders’ and followers’ perceptions may occur when leaders rate themselves as more rationally persuasive than their followers perceive. Followers can influence their leaders’ behavior by asking for more explanations, logical arguments and factual evidence to justify the request for certain tasks (Yukl, 2002). Furthermore, such situation may also be openly discussed during annual leader-follower development talks. Similarly to the fact that respectful leaders are considered to have high teachability (Owens et al., 2013), it is likely that leaders, who rate themselves as rational, are open to arguments and evidence from followers of the opposite. Therefore, these leaders may be likely to change their behavior over time. Yet, if a difference in perception persists, i.e., when the leader does not recognize or acknowledge (a lack in) their use of rational persuasion, we expect that this assessment gap may act as a resource passageway that negatively moderates the resilience-workability relationship. Resilient employees will be less inclined to invest their resources in improving their workability when they experience a gap between themselves and their leaders in the assessment of the use of rational persuasion by the leader.

In analogy with the situation in which pressure is being used, it may be the case that followers rate their leaders’ use of rational persuasion more positively than the leader does him/herself. In line with the notion of resources passageways, it is expected that the possibility to use followers’ resources (resilience) for obtaining more resources (workability) is complemented in these situations, as followers experience reasonable and justified (well-argued) requests from their leaders. Therefore, the positive resilience-workability relationship will be reinforced. Taken altogether, the following is hypothesized:

*Hypothesis 3*: Leader-employee differences in the assessment of leader rational persuasion moderates the positive relationship between resilience and workability in such a way that this relationship is buffered when follower ratings of leaders’ rational persuasion are lower than leader ratings, and strengthened when follower ratings are higher than leader ratings.

**Figure 1:**
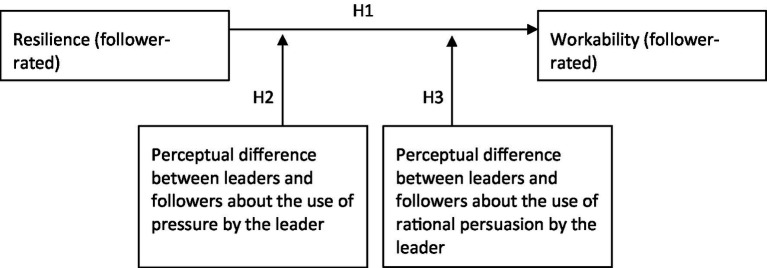
Conceptual model.

Figure 1 shows the conceptual model for this study.

## 3. Materials and methods

### 3.1. Sample

Data were collected *via* an online survey in two waves from 146 Dutch leader-follower dyads. In the first wave (W1), In the first wave (W1) data were gathered about the influence tactics used by the leader (leader-rated as well as employee-rated). In this wave, also self-reported data were collected from employees about their resilience. In the second wave (W2), data were gathered about the dependent variable, namely workability (employee-rated). The study was approved by the Ethics Committee of the researcher’s university. Respondents provided informed consent and various procedures were employed to limit common method bias (ability to stop anytime, request for honest responses, etc.).

In total 222 leaders-follower dyads were invited to participate in the study. The scores of leaders and followers were matched by using tokens and pseudonimisation. After the second wave of data collection among leaders and followers the final sample consisted of 146 unique matched leader-follower pairs. In this dataset 54% of leaders and 44% of followers was male. On average leaders were 46.1 years old (SD = 9.1), and followers 41.6 years (SD = 10.7). Furthermore, 88% of leaders and 67% of followers had a bachelor degree or higher.

### 3.2. Measures

Study variables were assessed using validated scales from prior studies.

#### 3.2.1. Employee resilience

Resilience was measured by the nine-item resilience at work scale from Näswall et al. (2019). This scale is explicitly designed to measure resilience capacity in workplace settings (Näswall et al., 2019). Example items are: “I resolve crises competently at work” and “I effectively respond to feedback at work, even criticism.” Following recommendations of Geldhof et al. (2014) and Cortina et al. (2020), we used McDonald’s (1999) omega (ω) to establish internal reliability of the scales, instead of the less accurate Cronbach’s alpha. Drawbacks from Cronbach’s alpha as compared to other indicators of reliability can be found, among others in Zinbarg et al. (2005), Revelle and Zinbarg (2009) and Cho and Kim (2015). The reliability analysis showed good internal reliability of the scale (ω = 0.80). Similar to Cronbach’s alpha, McDonald’s omega has a cutoff value of 0.7.

#### 3.2.2. Influence tactics

The influence tactics pressure and rational persuasion were each assessed with a four-item scale developed by Yukl et al. (2008). The influence tactics were assessed by leaders as well as followers, i.e., both dyad partners. Leaders rated themselves with respect to their use of pressure and their use of rational persuasion and followers rated the extent in which they perceived pressure (and rational persuasion) from their leader. Two example items for follower-rated pressure are: “My leader repeatedly checks to see whether I have carried out a request” and “My leader tries to pressure me to carry out a request” (*ω* = 0.69). We employed a referent-shift method (Chan, 1998) to ask the same items to leaders: “I repeatedly check to see whether employee X has carried out a request” and “I try to pressure X to carry out a request” (*ω* = 0.69). Two example items for follower-rated rational persuasion are: “My leader explains clearly why a proposed change is necessary to accomplish task objectives,” and “My leader uses facts and logic to make a persuasive case for a request or proposal” (*ω* = 0.87). Employing the referent-shift method leads to similar versions for the leader: “I explain clearly why a proposed change is necessary to accomplish task objectives” and “I use facts and logic to make a persuasive case for a request or proposal” (*ω* = 0.83). We constructed a new variable for the difference in leader-follower assessment of each influence tactic by subtracting the average follower rating from the average leader rating. Positive values therefore indicate that the leader rates him/herself higher on the use of a certain influence tactic than the follower.

#### 3.2.3. Workability

Workability was measured with the one-item dimension of the validated shortened Dutch version of the Workability Index (Ilmarinen et al., 2005). The item requires respondents to rate their current workability on a 1–10 rating scale. The use of single-item measures has been extensively discussed in the literature (e.g., Allen et al., 2022; Matthews et al., 2022) and it has been established that single-item measures display ample validity when constructs are unidimensional, well-defined, and narrow in scope (Allen et al., 2022; Matthews et al., 2022), as is the case for workability.

#### 3.2.4. Control variables

We controlled for demographic variables, including age, gender and education level as is the custom in studies about resilience and workability (e.g., Näswall et al., 2019). Age was measured in years. Gender was coded 0 for male and 1 for female. Education level was evaluated using six levels common to the Dutch educational system (1 = basic education; 2 = high school; 3 = applied education; 4 = higher applied education; 5 = university degree; 6 = PhD).

### 3.3. Analytical strategy

Analyses were performed using Jamovi open source software (The Jamovi Project, 2021), which uses R (R Core Team, 2021), as well as PROCESS for R (version 4.0.1). Collinearity statistics for the independent variables showed that all Variance Inflated Factors (VIFs) were below the recommended threshold of four (Hair et al., 2017), with the highest VIF being 1.06. Using a score-type variable for the leader-follower perception differences, limits the possibilities for testing convergent discriminant validity of the entire model. When assessing model fit by means of a Confirmatory Factor Analysis (CFA), including all items of resilience, leader-rated influence tactics and follower-rated influence tactics in a one-factor model generated the following fit measures: *χ*^2^ = 985; df = 275; RMSEA = 0.096; CFI = 0.46; TLI = 0.41. The CFA for the five factor-model showed that all items loaded on the intended factors, as is reflected in the fit statistics: *χ*^2^ = 381; df = 265; RMSEA = 0.039; CFI = 0.91; TLI = 0.90). Altogether, the CFAs indicated that the five-factor structure has a better fit than the alternative, one-factor model specification.

After performing a regression analysis, two moderation models were analyzed, using 10,000 bootstrap samples. Predictor variables were mean-centered before the analysis to enhance the interpretability of the moderation analyses. The first moderation model pertains to the moderation of the resilience-workability relationship by differences in assessment of leader pressure. The second moderation model applies the assessment of leader rational persuasion as a moderator in the resilience-workability relationship.

## 4. Results

The correlation matrix in Table 1 summarizes means, standard deviations and correlations between the main variables in our study. Table 1 shows that resilience at wave 1 (W1) is positively associated with workability at wave 2 (W2). Furthermore, the leader’s use of rational persuasion in the perception of the follower is positively related to the leader’s own rating of the degree to which this influence tactic is used, suggesting that leaders and followers align in their perspective. Contrastingly, the leader’s own rating of the use of pressure is not related to the perception of the follower about the extent to which the leader uses this influence tactic. This finding suggests that leaders and followers may differ in their assessment of the leader’s use of pressure. Interestingly, the mean of leader-rated use of pressure is higher than the mean ratings made by followers, suggesting that on average followers perceive less pressure than leaders perceive to exercise.

**Table 1:** Correlation matrix.

	Mean	SD	1	2	3	4	5	6	7	8	9	10	11	12	13	14
1. Workability (follower-rated; W2)	7.82	1.3	—													
2. Resilience (follower-rated; W1)	4.14	0.46	0.21***	(0.80)												
3. Perceptual difference about the use of pressure (DPRES)	0.38	0.69	0.08	0.17*	—											
4. Perceptual difference about the use of rational persuasion (DRATP)	0.03	0.78	0.01	−0.15	0.15	—										
5. Use of pressure (leader-rated, W1)	2.02	0.53	0.02	0.06	0.70***	0.12	(0.69)									
6. Use of pressure (follower-rated, W1)	1.65	0.49	−0.04	−0.14	−0.64***	−0.08	0.11	(0.69)								
7. Use of rational persuasion (leader-rated, W1)	4.00	0.6	−0.05	0.13	0.11	0.57***	0.10	−0.04	(0.83)							
8. Use of rational persuasion (follower-rated, W1)	3.95	0.68	−0.07	0.31***	−0.07	−0.68***	−0.04	0.06	0.22**	(0.87)						
9. Gender (leader)	0.46	0.5	−0.04	0.02	−0.04	−0.10	−0.15	−0.08	−0.13	−0.01	—					
10. Gender (follower)	0.56	0.5	−0.10	0.14*	−0.04	0.03	−0.03	−0.01	0.02	0.02	0.28***	—				
11. Education level (leader)	4.36	0.86	0.06	0.02	−0.05	−0.06	−0.03	0.03	0.14	0.16	0.06	0.08	—			
12. Education level (follower)	3.93	0.93	0.07	0.04	−0.07	0.15	−0.10	−0.05	0.18*	−0.03	−0.03	−0.02	0.14*	—		
13. Age (leader)	46.1	9.11	0.04	−0.06	0.06	0.14	−0.04	−0.07	0.04	−0.11	−0.09	0.06	0.03	0.11	—	
14. Age (follower)	41.6	10.7	0.02	−0.12	0.24**	0.08	0.15	−0.14	0.08	−0.03	−0.04	−0.10	0.02	−0.12	0.25***	—

*p < 0.05, **p < 0.01, ***p < 0.001; SD denotes the standard deviation; McDonald’s omega is displayed between brackets on the diagonal.

The correlations between the study variables and the demographic control variables provide the following insights. The education level of the follower is positively and significantly related to the use of persuasion of the leader (*r* = 0.18). This relationship could signal that highly educated followers are more likely to be encountered with rational persuasion than lower educated followers. Yet, the coefficient is rather small (well below 0.3, which is a commonly used threshold, e.g., Caniëls et al., 2022). Furthermore, Table 1 shows that age and gender of the follower are significantly associated with the perceptual difference between leaders and followers about the use of pressure (DPRES). Given the fact that DPRES concerns the discrepancy in perception between leader and follower it is difficult to assess whose gender/age (that of the follower or that of the leader) is showing the association. When observing the follower ratings and leader ratings (columns 5 and 6 in Table 1), no significant association between age/gender and the use of pressure can be identified. Taken together with the argument for parsimonious study designs and exclusion of impotent control variables (Becker, 2005; Bernerth and Aguinis, 2016), these controls were left out of further analysis to increase its power. Results from the regression and moderation analyses are shown in Table 2. As expected, resilience is shown to be positively and significantly related to workability, supporting hypothesis 1. The results further indicate a significant interaction between resilience and a leader-follower perceptual difference about the use of pressure, which is supportive of hypothesis 2. The interaction between resilience and a leader-follower perceptual difference about the use of rational persuasion is not significant. Hence, hypothesis 3 is not supported.

**Table 2:** Moderation analysis with workability as dependent variable (employee-rated; W2; *n* = 146).

	Regression model	Moderation Model 1 Pressure	Moderation Model 2 Rational Persuasion
Intercept	5.024	7.840	7.787
Resilience (Employee-rated; W1)	0.662*	0.694**	0.675**
Perceptual difference about the use of pressure (DPRES)	0.075	0.112	
Perceptual difference about the use of rational persuasion (DRATP)	0.068		0.069
Interaction (Resilience * DPRES)		−0.908*	
Interaction (Resilience * DRATP)			−0.153
*R* ^2^	0.053	0.083	0.053
*F*	2.63	4.29	2.63

**p < 0.01; *p < 0.05.

**Figure 2:**
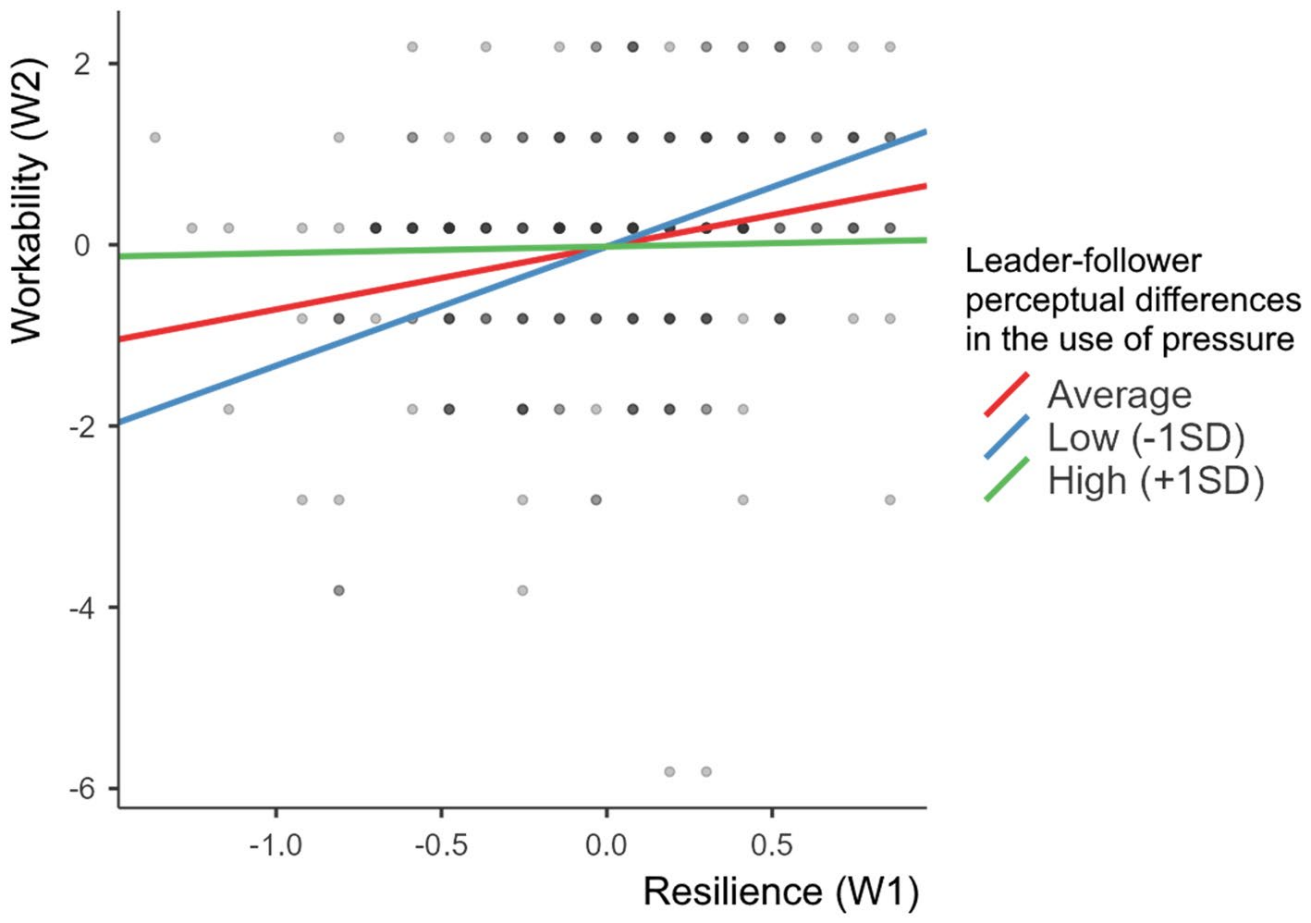
Simple slope analysis for the moderating effect of perceptual differences about the use of pressure on the resilience-workability relationship.

To analyse the significant moderation effect of perceptual differences about the use of pressure, a simple slope analysis was performed. Following the procedure suggested by Aiken and West (1991), simple slopes were tested for high (one standard deviation above the mean), moderate (mean) and low (one standard deviation below the mean) levels of the moderator. The significant interaction was plotted in Figure 2 using The Jamovi Project (2021). Figure 2 shows that the green (high moderator level; *b* = 0.07) line is less steep than the red (average moderator level; *b* = 0.69), indicating that high perceptual differences between leaders and followers concerning the leader’s use of pressure undermines the resilience-workability relationship. Additionally, the blue line is steeper (*b* = 1.31) than the red line (b = 0.69), indicating that low perceptual differences between leaders and followers concerning the leader’s use of pressure strengthens the resilience-workability relationship. The slopes of the blue and red lines significantly differ from zero. The slope of the green line does not significantly differ from zero. This pattern of results is in line with what was expected in hypothesis 2 and suggests the presence of a resource passageway.

## 5. Discussion

Most studies have explored leadership as an essential supportive (or hindering) factor that shapes the performance of employees. The present study assessed a dyadic perspective on the use of influence tactics by the leader. By investigating this issue, this study is one of the first to explore the boundary conditions that determine whether and how resilient behavior of employees is related to their workability. We found that leader-follower differences in perception about the use of pressure as an influence tactic moderates the resilience-workability relationship of followers. Where on average resilient employees possess resources that are associated with a high workability, this positive relationship is buffered when followers experience more pressure from their leader than the leader is thinking that he/she exerts on the follower. We did not find support for a similar effect with regard to rational persuasion.

The findings of this study have several theoretical and practical implications. First, our study contributes to COR theory by investigating a relatively less examined element of COR theory, namely the role of resource passageways (Halbesleben et al., 2014). Resource passageways are shaped by organizational circumstances that may stimulate the creation and maintenance of resources when they are favorable or may deplete resources when they are stacked against employees (Hobfoll, 2014). In this study, leader-follower assessment differences regarding the leader’s use of pressure as an influence tactic have been found to hamper the conservation of resources (specifically, resilience) by followers. The assessment difference adds an extra load on followers, who try to conserve their scarce resources, and such differences buffer a positive resilience-workability relationship. In other words, assessment differences concerning the use of pressure suppress the utilization of resilience by followers and its passage to workability. As predicted by COR theory, and consistent with the notion of resource passageways (Halbesleben et al., 2014), resource gains from resilience in the form of increased workability are overshadowed by the larger influence of resource loss as a consequence of a pressuring leader who does not fully recognize his/her pressuring influence. Evidence for this effect is indicated by the way in which the red line in Figure 2 transforms into the green line and becomes less steep when followers rate the utilization of pressure higher than their leaders. These results tie in with prior studies about the boundary condition asserted by a safety climate and studies on how resources function (e.g., Loh et al., 2018). The findings are also in line with leadership research that explores the importance of follower perceptions of interpersonal leadership (Aslam et al., 2021) and the leader-member exchange relationship (for a review, see Epitropaki and Martin, 2016) in accelerating (or hindering) employees’ investment of energy and resources in performance behaviors. When employees entertain positive perceptions of interpersonal leadership and of their personal leader-member exchange relationship, they develop a sense of psychological safety that is conducive to an initiative climate, i.e., a climate in which employees are self-starting, oriented at improvements, persistent and long-term-focused (Aslam et al., 2021).

Second, this study advances current insights into the impact of differences in perspective between leaders and followers about the degree in which certain influence tactics are used. We found no effect of leader-follower assessment differences regarding rational persuasion, which may be explained by the notion that rational persuasion appeals to the ratio of followers, whereas pressure may have an effect on a more subconscious level. Leaders who use rational persuasion to a larger extent than they acknowledge are at the root of a leader-follower assessment difference, but the findings indicate that this difference does not influence the resilience-workability relationship. In contrast, influencing followers by pressuring them depletes their resources and leader-follower assessment differences in the use of pressure counteracts the resource gain process that on average exists between resilience and workability. When followers feel pressure (unacknowledged by their leader), they hold close to their resources (resilience) and do not invest them into workability. Apparently, perceptual differences about pressure are more damaging to resource development than perceptual differences about persuasion. Unacknowledged pressure of the leader can induce feelings of hopelessness in the follower, as the situation is not likely to change any time soon. This realization may therefore have a resource depleting effect. Studies about destructive leadership (Trépanier et al., 2013) and dark triad character traits of leaders (Volmer et al., 2016) have shown how pressure from leaders, especially when it cannot be addressed, undermines followers’ motivation and drains their personal resources and can even jeopardize their health (Kile, 1990). In contrast, unacknowledged (lack of) the use of persuasion as an influence tactic by leaders may feel different to followers, as leaders who claim to be rational may do their best in explaining their motivations for their task requests to followers. By asking for more clarification and underlying reasons for the task request, followers may satisfy their need for arguments to justify the request for certain tasks (Yukl, 2002). In other words, followers have the means to address the perceived lack of using persuasion by their leaders. Perceptual gaps between leaders and followers about the leaders’ use of rational persuasion can therefore be overcome.

### 5.1. Practical implications

The study’s findings suggest that leader-follower assessment differences with regard to the leader’s use of pressure served as a resource passageway, draining resources, resulting in a buffered relation between followers’ resilience and workability. This study’s results suggest that organizations can improve the resilience-workability relationship by curbing leader-follower assessment differences of influence tactics (specifically, the utilization of pressure by the leader). Organizations are advised to undertake efforts to improve the self-awareness of leaders. Here could lie a prominent role for human resource management practices, as targeted trainings may be beneficial in this respect. It has been shown that training leaders in self-awareness is positively associated with follower-rated transformational leadership (Cerni et al., 2010). Therefore, self-awareness trainings are expected to diminish the gap between leaders’ and followers’ perception of leaders’ utilization of pressure, which may in turn lift its buffering effect on the followers’ resilience-workability relationship.

Furthermore, the study provided evidence of a positive association between resilience and workability of employees. Therefore, organizations may want to direct resources toward improving employee resilience. Previous studies have posed several concrete ways in which resilience among workers can be stimulated (Bardoel et al., 2014; Caniëls and Hatak, 2019). Human resource practices that are oriented at personal development and growth can be offered to employees in order to strengthen their resilience and thereby their workability. For example, specifically designed interventions may encourage and foster resilience qualities among employees (Bardoel et al., 2014). In addition, organizations can provide adequate tools to buffer the depletion of resources as much as possible. Not only self-awareness trainings for leaders that diminish perception gaps can help in this respect, but also organizational practices that curtail the depletion of resources in general. For example, it has been shown that when employees are given a degree of control over their work that matches their needs, this job control acts as a buffer against depletion of resources (Parker et al., 2010).

### 5.2. Limitations and future research

Several limitations have to be taken into account when interpreting this study. Firstly, we adopted a two-wave design to test a moderation model. Although a two-wave study is preferred over a cross-sectional design, we can merely offer evidence of causality, but we cannot provide absolute proof. Future studies may want to test the robustness of our proposed model by correcting for auto-correlations in the dependent variable.

Secondly, although evaluating leader-follower perception differences in the leader’s use of influence tactics is new and original, we were limited in that we tested only two influence tactics, namely pressure and rational persuasion. Yukl et al. (2008) have identified a wide range of possible tactics that can be used in the workplace. Future studies may broaden the set of influence tactics under study. Even though studies are known to use subsets of influence tactics (e.g., Sparrowe et al., 2006), it may be especially interesting for future research to design studies that incorporate all tactics.

Thirdly, as was pointed out by one of the anonymous reviewers, Hobfoll et al. (2018) proposes that resource spirals are more rigid than loss spirals, i.e., the progression on a loss spiral is more swift than the progression on a resource spiral. The current study did only explore whether a resource passageway could be established at all. Future studies may have *a priori* hypotheses about the relative strength of a loss spiral versus a resource/gain spiral. Testing such hypotheses would require multiple (more than two) measurements of this study’s variables.

Lastly, although multiple sources (leaders and followers) were used for this study, the antecedent and outcome variables were rated by followers, while only moderating variables were rated by both, followers and leaders. This procedure may have introduced common method bias to the study. Given that resilience and workability are difficult to assess by others than oneself, because external raters often fall back on general impressions (Lance et al., 1994), the use of self-reports in such cases is generally warranted (Conway and Lance, 2010). Furthermore, we followed recommendations of Podsakoff et al. (2012) about curbing method biases through survey design, by measuring antecedent and outcome variables at different points in time. Therefore, given the nature of the study variables, as well as the multiwave design of the study, risks of common method bias are low. Nevertheless, future studies may pursue alternative research designs to further curtail the risk of bias. Relatedly, due to the fact that the study used multiple waves (wave 1 and wave 2) and multiple sources (leaders and followers), the final size of the sample is quite small (*n* = 146), which may have consequences for the accuracy and reliability of the estimates (Shadish et al., 2002). This is the price paid for the rigorous study design that allows for a time lag between the measurement of the dependent and independent variables, thereby following Podsakoff et al.’s (2003) recommendations for limiting the risk on method biases through study design. Future studies are advised to check the robustness of this study’s model in larger samples.

Notwithstanding these limitations, the present study and its findings has advanced current insights about how the resilience-workability relationship can be influenced by leader-follower differences in assessment of the leader’s use of influence tactics.

## Data availability statement

The datasets presented in this article are not readily available because respondents have not consented to sharing the data. Requests to access the datasets should be directed to MC, marjolein.caniels@ou.nl.

## Ethics statement

The studies involving human participants were reviewed and approved by CETO (Commissie Ethische Toetsing) of the Open Universiteit Open Universiteit, Heerlen, Netherlands. The patients/participants provided their written informed consent to participate in this study.

## Author contributions

MC gathered the data, performed the analyses, and wrote the paper.

## Conflict of interest

The author declares that the research was conducted in the absence of any commercial or financial relationships that could be construed as a potential conflict of interest.

## Publisher’s note

All claims expressed in this article are solely those of the authors and do not necessarily represent those of their affiliated organizations, or those of the publisher, the editors and the reviewers. Any product that may be evaluated in this article, or claim that may be made by its manufacturer, is not guaranteed or endorsed by the publisher.

## References

[ref600] Aiken L. S. West S. G. (1991). Multiple regression: Testing and interpreting interactions. Newbury Park, London: Sage.

[ref1] Allen M. S. Iliescu D. Greiff S. (2022). Single item measures in psychological science. Eur. J. Psychol. Assess. 38, 1–5. doi: 10.1027/1015-5759/a000699

[ref2] Aslam M. Z. Fateh A. Omar S. Nazri M. (2021). The role of initiative climate as a resource caravan passageway in developing proactive service performance. Asia Pac. J. Bus. Adm. 14, 691–705. doi: 10.1108/APJBA-09-2021-0454

[ref3] Avey J. B. Luthans F. Jensen S. M. (2009). Psychological capital: a positive resource for combating employee stress and turnover. Hum. Resour. Manag. 48, 677–693. doi: 10.1002/hrm.20294

[ref4] Avey J. B. Reichard R. J. Luthans F. Mhatre K. M. (2011). Meta-analysis of the impact of positive psychological capital on employees’ attitudes, behaviors, and performance. Hum. Resour. Dev. Q. 22, 127–152. doi: 10.1002/hrdq.20070

[ref5] Bardoel E. A. Pettit T. M. De Cieri H. McMillan L. (2014). Employee resilience: an emerging challenge for HRM. Asia Pac. J. Hum. Resour. 52, 279–297. doi: 10.1111/1744-7941.12033

[ref6] Becker T. E. (2005). Potential problems in statistical control of variables in organizational research. Organ. Res. Methods 8, 274–289. doi: 10.1177/1094428105278021

[ref7] Bernerth J. B. Aguinis H. (2016). A critical review and best-practice recommendations for control variable usage. Pers. Psychol. 69, 229–283. doi: 10.1111/peps.12103

[ref8] Caniëls M. C. J. Baaten S. M. (2019). How a learning-oriented organizational climate is linked to different proactive behaviors: the role of employee resilience. Soc. Indic. Res. 143, 561–577. doi: 10.1007/s11205-018-1996-y

[ref9] Caniëls M. C. J. Hatak I. (2019). Employee resilience: considering both the social side and the economic side of leader-follower exchanges in conjunction with the dark side of followers’ personality. Int. J. Human Resour. Manag. 33, 297–328. doi: 10.1080/09585192.2019.1695648

[ref10] Caniëls M. C. J. Nikolova I. Hatak I. de Weerd-Nederhof P. C. (2022). Antecedents of COVID-19 rumination: a three-wave study. Scand. J. Psychol. 63, 476–483. doi: 10.1111/SJOP.12832, PMID: 35604020 PMC9347792

[ref11] Cerni T. Curtis G. J. Colmar S. H. (2010). Increasing transformational leadership by developing leaders’ information-processing systems. J. Leadersh. Stud. 4, 51–65. doi: 10.1002/jls.20177

[ref12] Chan D. (1998). Functional relations among constructs in the same content domain at different levels of analysis: a typology of composition models. J. Appl. Psychol. 83, 234–246. doi: 10.1037/0021-9010.83.2.234

[ref13] Cho E. Kim S. (2015). Cronbach’s coefficient alpha: well known but poorly understood. Organ. Res. Methods 18, 207–230. doi: 10.1177/1094428114555994

[ref14] Clarke S. Ward K. (2006). The role of leader influence tactics and safety climate in engaging employees’ safety participation. Risk Anal. 26, 1175–1185. doi: 10.1111/j.1539-6924.2006.00824.x, PMID: 17054524

[ref15] Conway J. M. Lance C. E. (2010). What reviewers should expect from authors regarding common method bias in organizational research. J. Bus. Psychol. 25, 325–334. doi: 10.1007/s10869-010-9181-6

[ref16] Cortina J. M. Sheng Z. Keener S. K. Keeler K. R. Grubb L. K. Schmitt N.. (2020). From alpha to omega and beyond! A look at the past, present, and (possible) future of psychometric soundness in the journal of applied psychology. J. Appl. Psychol. 105, 1351–1381. doi: 10.1037/apl0000815, PMID: 32772525

[ref17] Coutu D. L. (2002). How resilience works. Harv. Bus. Rev. 80, 46–50. 12024758

[ref18] Curtis G. J. (2018). Connecting influence tactics with full-range leadership styles. Leadersh. Organ. Dev. J. 39, 2–13. doi: 10.1108/LODJ-09-2016-0221

[ref19] Epitropaki O. Martin R. (2016). “LMX and work attitudes: is there anything left unsaid or unexamined?” in The Oxford Handbook of Leader-Member Exchange. eds. Bauer T. N. Erdogan B. E. (New York, NY: Oxford University Press), 157–174.

[ref20] Geldhof G. J. Preacher K. J. Zyphur M. J. (2014). Reliability estimation in a multilevel confirmatory factor analysis framework. Psychol. Methods 19, 72–91. doi: 10.1037/a0032138, PMID: 23646988

[ref21] Gómez-Molinero R. Ruiz-González P. Zayas A. Gil R. (2019). Resilience and workability among breast cancer survivors. INFAD (Barcelona) 4, 37–44. doi: 10.17060/ijodaep.2019.n1.v4.1503

[ref22] Hair J. Hollingsworth C. L. Randolph A. B. Chong A. Y. L. (2017). An updated and expanded assessment of PLS-SEM in information systems research. Ind. Manag. Data Syst. 117, 442–458. doi: 10.1108/IMDS-04-2016-0130

[ref23] Halbesleben J. R. Neveu J. P. Paustian-Underdahl S. C. Westman M. (2014). Getting to the ‘COR’: understanding the role of resources in conservation of resources theory. J. Manag. 40, 1334–1364. doi: 10.1177/0149206314527130

[ref24] Hobfoll S. E. (1989). Conservation of resources: a new attempt at conceptualizing stress. Am. Psychol. 44, 513–524. doi: 10.1037/0003-066X.44.3.513, PMID: 2648906

[ref100] Hobfoll S. E. (2001). The influence of culture, community, and the nested self in the stress process: Advancing conservation of resources theory. Appl. Psychol Int. Rev. 50, 337–370.

[ref25] Hobfoll S. E. (2011). Conservation of resource caravans and engaged settings. J. Occup. Organ. Psychol. 84, 116–122. doi: 10.1111/j.2044-8325.2010.02016.x

[ref26] Hobfoll S. E. (2012). Conservation of resources and disaster in cultural context: the caravans and passageways for resources. Psychiatry 75, 227–232. doi: 10.1521/psyc.2012.75.3.227, PMID: 22913498

[ref300] Hobfoll S. E. (2014). Resource caravans and resource caravan passageways: A new paradigm for trauma responding. Intervention (Amstelveen, Netherlands) 12, 21–32. doi: 10.1097/WTF.0000000000000067

[ref200] Hobfoll S. E. Halbesleben J. Neveu J.-P. Westman M. (2018). Conservation of resources in the organizational context: the reality of resources and their consequences. Annu. Rev. Organ. Psychol. Organ. Behav. 5, 103–128.

[ref27] Ilmarinen J. Tuomi K. Seitsamo J. (2005). New dimensions of work ability. Int. Congr. Ser. 1280, 3–7. doi: 10.1016/j.ics.2005.02.060

[ref28] Kacmar K. M. Carlson D. S. Harris K. J. (2013). Interactive effect of leaders’ influence tactics and ethical leadership on work effort and helping behavior. J. Soc. Psychol. 153, 577–597. doi: 10.1080/00224545.2013.798248, PMID: 24003584

[ref29] Kane K. Montgomery K. (1998). A framework for understanding dysempowerment inorganizations. Hum. Resour. Manag. 37, 263–275. doi: 10.1002/(SICI)1099-050X(199823/24)37:3/4<263::AID-HRM8>3.0.CO;2-U

[ref30] Katz D. Kahn R. L. (1966). The Social Psychology of Organizations. New York, NY: Wiley.

[ref31] Kile S. M. (1990). Helsefarleg Leierskap (Health Endangering Leadership); Oslo, Norway: Hjemmets Bokforlag.

[ref32] Kumar K. Beyerlein M. (1991). Construction and validation of an instrument for measuring ingratiatory behaviors in organizational settings. J. Appl. Psychol. 76, 619–627. doi: 10.1037/0021-9010.76.5.619

[ref33] Kuntz J. C. Näswall K. Malinen S. (2016). Resilient employees in resilient organizations: flourishing beyond adversity. Indus. Organ. Psychol. Perspect. Sci. Pract. 9, 456–462. doi: 10.1017/iop.2016.39

[ref34] Lance C. E. LaPointe J. A. Fisicaro S. A. (1994). Test of three causal models of halo rater error. Organ. Behav. Hum. Decis. Process. 57, 83–96. doi: 10.1006/obhd.1994.1005

[ref35] Lodi-Smith J. Roberts B. W. (2007). Social investment and personality: a meta-analysis of the relationship of personality traits to investment in work, family, religion, and volunteerism. Personal. Soc. Psychol. Rev. 11, 68–86. doi: 10.1177/1088868306294590, PMID: 18453456

[ref36] Loh M. Y. Idris M. A. Dollard M. F. Isahak M. (2018). Psychosocial safety climate as a moderator of the moderators: contextualizing JDR models and emotional demands effects. J. Occup. Organ. Psychol. 91, 620–644. doi: 10.1111/joop.12211

[ref37] Luthans F. (2002). Positive organizational behavior: developing and managing psychological strengths. Acad. Manag. Perspect. 16, 57–72. doi: 10.5465/ame.2002.6640181

[ref500] Luthans F. Avolio B. J. Avey J. B. Norman S. M. (2007). Positive psychological capital: Measurement and relationship with performance and satisfaction. Pers. Psychol. 60, 541–572. doi: 10.1111/j.1744-6570.2007.00083.x

[ref38] Matthews R. A. Pineault L. Hong Y. (2022). Normalizing the use of single-item measures. J. Bus. Psychol. 37, 639–673. doi: 10.1007/s10869-022-09813-3

[ref39] McDonald R. P. (1999). Test Theory: A Unified Treatment. Mahwah, NJ: Erlbaum.

[ref40] Meneghel I. Salanova M. Martínez I. M. (2016). Feeling good makes us stronger. J. Happiness Stud. 17, 239–255. doi: 10.1007/s10902-014-9592-6

[ref41] Näswall K. Malinen S. Kuntz J. Hodliffe M. (2019). Employee resilience: development and validation of a measure. J. Manag. Psychol. 34, 353–367. doi: 10.1108/JMP-02-2018-0102

[ref42] Owens B. P. Johnson M. D. Mitchell T. R. (2013). Expressed humility in organizations: implications for performance, teams, and leadership. Organ. Sci. 24, 1517–1538. doi: 10.1287/orsc.1120.0795

[ref43] Papazoglou K. Andersen J. P. (2014). A guide to utilizing police training as a tool to promote resilience and improve health outcomes among police officers. Traumatology 20, 103–111. doi: 10.1037/h0099394

[ref44] Parker S. L. Jimmieson N. L. Amiot C. E. (2010). Self-determination as a moderator of demands and control: implications for employee strain and engagement. J. Vocat. Behav. 76, 52–67. doi: 10.1016/j.jvb.2009.06.010

[ref45] Paterson T. A. Luthans F. Jeung W. (2014). Thriving at work: impact of psychological capital and supervisor support. J. Organ. Behav. 35, 434–446. doi: 10.1002/job.1907

[ref46] Peterson S. J. Luthans F. Avolio B. J. Walumbwa F. O. Zhang Z. (2011). Psychological capital and employee performance: a latent growth modeling approach. Pers. Psychol. 64, 427–450. doi: 10.1111/j.1744-6570.2011.01215.x

[ref47] Podsakoff P. M. MacKenzie S. B. Lee J.-Y. Podsakoff N. P. (2003). Common method biases in behavioral research: a critical review of the literature and recommended remedies. J. Appl. Psychol. 88, 879–903. doi: 10.1037/0021-9010.88.5.879, PMID: 14516251

[ref48] Podsakoff P. M. MacKenzie S. B. Podsakoff N. P. (2012). Sources of method bias in social science research and recommendations on how to control it. Annu. Rev. Psychol. 63, 539–569. doi: 10.1146/annurev-psych-120710-100452 21838546

[ref49] R Core Team (2021). R: A Language and Environment for Statistical Computing. (version 4.0) [computer software]. Available at: https://cran.r-project.org (Accessed December 22, 2022).

[ref50] Revelle W. Zinbarg R. E. (2009). Coefficients alpha, Beta, omega, and the glb: comments on Sijtsma. Psychometrika 74, 145–154. doi: 10.1007/s11336-008-9102-z

[ref51] Roberts B. W. Wood D. (2006). “Personality development in the context of the neo-Socioanalytic model of personality,” in Handbook of Personality Development. eds. Mroczek D. K. Little T. D. (Mahwah, NJ: Lawrence Erlbaum Associates Publishers), 11–39.

[ref52] Roche M. Haar J. M. Luthans F. (2014). The role of mindfulness and psychological capital on the well-being of leaders. J. Occup. Health Psychol. 19, 476–489. doi: 10.1037/a0037183, PMID: 24933594

[ref53] Semeijn J. H. Caniëls M. C. J. Kooistra D. (2019). Cross-lagged effects of resilience and indicators of sustainable employability; a study among Dutch police officers. Policing: an. Int. J. 42, 961–975. doi: 10.1108/PIJPSM-01-2019-0003

[ref54] Shadish W. R. Cook T. D. Campbell D. T. (2002). Experimental and Quasi-Experimental Designs for Generalized Causal Inference. Boston, MA: Houghton Mifflin Company

[ref55] Siu O. L. Cheung F. Lui S. (2015). Linking positive emotions to work well-being and turnover intention among Hong Kong police officers: the role of psychological capital. J. Happiness Stud. 16, 367–380. doi: 10.1007/s10902-014-9513-8

[ref56] Smith B. W. Dalen J. Wiggins K. Tooley E. Christopher P. Bernard P. (2008). The brief resilience scale: assessing the ability to bounce back. Int. J. Behav. Med. 15, 194–200. doi: 10.1080/10705500802222972, PMID: 18696313

[ref57] Sparrowe R. T. Soetjipto B. W. Kraimer M. L. (2006). Do leaders’ influence tactics relate to members’ helping behavior? It depends on the quality of the relationship. Acad. Manag. J. 49, 1194–1208. doi: 10.5465/AMJ.2006.23478645

[ref58] The Jamovi Project (2021). Jamovi. (version 2.2) [computer software]. Available at: https://www.jamovi.org (Accessed December 22, 2022).

[ref59] Trépanier S. Fernet C. Austin S. (2013). Workplace bullying and psychological health at work: the mediating role of satisfaction of needs for autonomy, competence and relatedness. Work Stress. 27, 123–140. doi: 10.1080/02678373.2013.782158

[ref60] Upadyaya K. Vartiainen M. Salmela-Aro K. (2016). From job demands and resources to work engagement, burnout, life satisfaction, depressive symptoms, and occupational health. Burn. Res. 3, 101–108. doi: 10.1016/j.burn.2016.10.001

[ref61] Van den Berg T. I. Elders L. A. de Zwart B. C. Burdorf A. (2009). The effects of work-related and individual factors on the work ability index: a systematic review. Occup. Environ. Med. 66, 211–220. doi: 10.1136/oem.2008.039883, PMID: 19017690

[ref400] Van Dam P. Shannon E. A. (2013). Developing positive leadership in health and human services: Original research. SA J. Ind. Psychol. 39, 1–11.

[ref62] Volmer J. Koch I. K. Göritz A. S. (2016). The bright and dark sides of leaders’ dark triad traits: effects on subordinates’ career success and well-being. Pers. Individ. Differ. 101, 413–418. doi: 10.1016/j.paid.2016.06.046

[ref63] Wang Z. Li C. Li X. (2017). Resilience, leadership and work engagement: the mediating role of positive affect. Soc. Indic. Res. 132, 699–708. doi: 10.1007/s11205-016-1306-5./10.1177/0149206307305562

[ref64] Wong S. I. (2019). Influencing upward: Subordinates’ responses to leaders’ (un)awareness of their empowerment expectations. Int. J. Hum. Resour. Manag. 30, 1604–1634. doi: 10.1080/09585192.2017.1299194

[ref65] Xanthopoulou D. Bakker A. B. Demerouti E. Schaufeli W. B. (2009). Reciprocal relationships between job resources, personal resources, and work engagement. J. Vocat. Behav. 74, 235–244. doi: 10.1016/j.jvb.2008.11.003

[ref66] Youssef C. M. Luthans F. (2007). Positive organizational behavior in the workplace. J. Manag. 33, 774–800. doi: 10.1177/0149206307305562

[ref67] Yukl G. (2002). Leadership in Organizations (5th Edn). Upper Saddle River, NJ: Prentice-Hall.

[ref68] Yukl G. Chavez C. Seifert C. F. (2005). Assessing the construct validity and utility of two new influence tactics. J. Organ. Behav. 26, 705–725. doi: 10.1002/job.335

[ref69] Yukl G. Seifert C. F. Chavez C. (2008). Validation of the extended influence behavior questionnaire. Leadersh. Q. 19, 609–621. doi: 10.1016/j.leaqua.2008.07.006

[ref70] Zinbarg R. E. Revelle W. Yovel I. Li W. (2005). Cronbach’s alpha, Revelle’s, and McDonald’s omega H: their relations with each other and two alternative conceptualizations of reliability. Psychometrika 70, 123–133. doi: 10.1007/s11336-003-0974-7

